# Activist Burnout Among Climate Justice Activists in Austria: An Interpretative Phenomenological Analysis

**DOI:** 10.3390/healthcare13162045

**Published:** 2025-08-19

**Authors:** Gavin Lord, Hilda Reilly, Henriette Löffler-Stastka

**Affiliations:** 1 Postgraduate Unit, Medical University of Vienna, A-1090 Vienna, Austriahenriette.loeffler-stastka@meduniwien.ac.at (H.L.-S.); 2 Department of Psychoanalysis and Psychotherapy, Medical University Vienna, A-1090 Vienna, Austria

**Keywords:** activist burnout, activist persistence, social movements, sustainability, climate justice

## Abstract

**Background/Objectives**: Research shows that engaging in system-critical activism can be associated with negative mental health outcomes. This exploratory, idiographic, qualitative study seeks to develop an understanding of the experience of activist burnout among climate justice activists. It aims to answer the following research question: How do climate justice activists make sense of their activist burnout? **Methods**: An Interpretative Phenomenological Analysis was conducted using interviews with five participants (*n* = 5) who are part of climate justice movements in Austria. **Results**: Three themes were constructed: (1) “The earth is burning”—describing the sense of urgency and pressure experienced by participants; (2) “Relationships with activism, other activists, and the outside world”—describing the ways in which activism colors the participants’ relationship with themselves and others; and (3) “Burning out”—describing the experience of activist burnout at its most acute point. **Discussion and Implications**: A picture of activist burnout emerges that is characterized by a sense of existential crisis and lack of alternatives; activist work becomes all-consuming and the individual may become isolated. Eventually, activist work evokes stress and cynicism in the individual and may culminate in a breaking point. In many ways, this picture of activist burnout bears similarity to burnout among the helping professions. Implications and recommendations for activists, organizers, and researchers are given.

## 1. Introduction

Engaging in system-critical activism can bring with it a sense of community and empowerment [1]. However, it can also be associated with negative mental health outcomes. In one study on the mental health effects of student activism, 60% of respondents reported that their activism had adversely affected their psychological well-being [2]. In another qualitative study on burnout among social justice activists, participants attributed chronic, debilitating stress and depression, as well as serious somatic conditions such as cancer, to their engagement in activism [3].

The concept of burnout—as well as associated phenomena, such as “helper syndrome”—was originally described as an occupational hazard of the helping professions (e.g., doctors, psychologists, psychotherapists, educators, social workers) [4,5,6]. Beginning roughly in the 1990s; however, the concept of burnout became associated with a wider range of occupations [7]. The early literature on activist burnout more or less coincides with this expansion of the concept of burnout [8].

The phenomenon of activist burnout has been defined within the literature as a condition in which activists must scale-back or disengage from activism, at least temporarily, due to the long-term and accumulative impact of stress that they associate with their activism [3,9,10]. It has been estimated that activist burnout affects 50–60% of activists within social movements [11].

Activist burnout is linked with deleterious consequences for the physical and emotional well-being of activists [12,13]. Furthermore, activist burnout presents a formidable challenge to the sustainability of social movements, where success often hinges upon the persistence of particular, often long-standing, activists who play critical roles [13,14].

The existing literature has examined the activist burnout experiences of queer college activists of color [12], social justice activists [3,11], and racial justice activists [15]. Although there is literature on the factors that cause long-term environmental activists to burnout or to persist [13], the authors are not aware of any studies that examine the burnout experience of climate justice activists. Recent literature suggests that climate justice activism is associated with a complex range of emotions, such as climate anxiety and climate grief, as well as intensified feelings of hope, but also of despair [16]. Furthermore, increased engagement with information relating to climate change is negatively linked to mental well-being [17]. It therefore stands to reason that the experience of burnout among climate justice activists may have qualitative differences to activist burnout in other contexts.

Burnout is not generic—it is context- and occupation-specific, and effective interventions must be tailored to these specific needs [18]. Therefore, it is essential to develop an understanding of activist burnout within this specific context.

The goal of this research is to develop an understanding of the experience of activist burnout among climate justice activists. Rather than seeking to describe the etiology or symptoms of activist burnout, this research is concerned first and foremost with providing insights into the subjective experience of activist burnout amongst the participants. The research follows an exploratory, inductive, and idiographic approach, making use of Interpretative Phenomenological Analysis (IPA) to capture the complex lived experience of the participants.

This study seeks to answer the following research question: How do system-critical climate activists make sense of their activist burnout?

## 2. Methods

### 2.1. Interpretative Phenomenological Analysis

The qualitative method used in this study, Interpretative Phenomenological Analysis, is an inductive and idiographic method that examines the lived experience of the participant; specifically, what is an experience like for the participant [19,20]. IPA is particularly well-suited to exploratory research, as it can produce meaningful results with relatively small sample sizes [21]. As IPA is an inductive method that seeks to provide insight into participants’ lived experiences, it is not intended to test a pre-formulated hypothesis.

According to Smith et al. [22], accepted as the authoritative account of IPA, “the essence of IPA lies in its analytic focus” [22] (p. 80), which can be characterized by iterative movement within the hermeneutic circle. Within the context of the participant’s whole account, particularly prescient passages emerge. These passages are viewed in great detail, word-for-word, before being considered once again within the context of the whole. This process is described as a “dynamic, non-linear, style of thinking” [22] (p. 30)—the researcher moves back and forth through the analytic process, exploring different ways of thinking about the data, rather than from beginning to end in a strict, step-by-step fashion.

During the analysis, the researcher attempts both to place themselves within the participant’s shoes, while simultaneously taking a ‘questioning’ stance, where the researcher critically considers meanings that may not be immediately apparent [22] (p. 27).

Despite this non-linear and creative approach, Smith et al. [22] (p. 31) describe how IPA research is characterized by commitment to a set of strategies. These can be summarized as follows: a line-by-line analysis of the data through which themes are identified, which takes into consideration convergence and divergence within individual cases, as well as across multiple cases. An analytic structure is developed and woven into a narrative. During this process, the researcher reflects upon their own perceptions, usually with the help of supervision or colleagues. Finally, the research is presented in a manner that demonstrates plausibility and allows the reader insight into the analytic process [22] (p. 80).

The analysis takes place at four levels. Experiential Statements are the first layer. These are concise statements that capture a particular aspect of the participant’s lived experience, staying close to the participant’s own words. These are clustered or grouped into Personal Emergent Themes for each participant. Lastly, Group Emergent Themes are constructed at the group level in a manner that presents both convergence and divergence in the participants’ accounts [22]. As an example, an element of pressing urgency was present in the account of every participant, which became the Experiential Statement “Crisis” (described in Section 3.1.1). This Experiential Statement seemed to have something in common with the Experiential Statements “No alternative to activism” and “If not me, then who?”—all three relate to the impetus for the participants’ involvement in activism. Thus, they were grouped together under the Group Emergent Theme “The earth is burning”.

Reflexivity on behalf of the researcher is an essential component of IPA research [23]. In the present research, reflexivity was managed through discussions between the main researcher and the supervisors, as well as with professional colleagues in the psycho-social field. This input iteratively impacted the coding and interpretation of the interview data. For example, the researcher found that he tended to allow Beppo’s account to sink into the background, while Rose’s account more readily caught his attention. This was attributed to the “heavy” and slow manner in which Beppo spoke, which evoked a feeling of frustration in the researcher, in contrast with Rose’s rushed and seemingly anxious manner, which seemed to almost “engulf” the researcher.

As a professional working in the field of social pedagogy, the researcher is intimately familiar with the challenges of maintaining a healthy relationship with one’s work. To a limited extent, he was previously involved in climate justice activism. At times these personal experiences served as a resource, giving the researcher a degree of common ground with the participants. At other times it needed to be consciously bracketed, for example, in relation to the researcher’s own experiences with climate guilt.

### 2.2. Recruitment and Interview Process

Participant recruitment took place from June through September 2023. Participants were found through a combination of word-of-mouth in activist social circles, as well as by sending the recruitment flyer directly to climate justice social movements in Austria.

For inclusion in this research, the participants must have needed take a break or strongly reduce their involvement in activist work, at least temporarily, due to stress factors connected to their activism. As such, the research is not targeted towards individuals who have a clinical diagnosis.

Three scoping interviews were conducted, after which the interview questions were refined, after which the five participants in this study were interviewed. The sample size (*n* = 5) follows the saturation method and also the recommendation of Smith et al. [22] for Master’s-level IPA research and strikes a pragmatic balance in terms of the presentability of results.

Interviews were conducted in a semi-structured fashion, in the German language. Where necessary, the interviewer asked follow-up questions or encouraged participants to explain in more detail. Interviews lasted roughly 50 min and were recorded and stored offline on a secure device, after which they were transcribed. An English translation of the Interview Guide can be found in the Appendix A. Participant quotes were translated into English in the interest of readability.

Interview recordings and transcripts were anonymized using pseudonyms selected by the participants. All identifiable information has been removed from participant quotes used in the final presentation of the research. Ethical approval for the research was granted by the Ethics Committee of the Medical University of Vienna on 7 July 2023.

To control for interrater-reliability the process of coding was followed by discussions between the main researcher and the supervisors, as well as with professional colleagues in the psycho-social field. This input iteratively impacted the coding and interpretation of the interview data, helped building themes, and to agree on the saturation of the themes within the material and the number of participants. Of course, a participative member check or triangulation was not applied, which might be seen as a limitation in qualitative research [24].

### 2.3. Participant Overview

Lilly (she/her) is in her early thirties, describes herself as European, and currently lives off of a student stipend. She has been involved in antifascist activism for 20 years and climate justice activism for 7 years.

Hazel (they/them) is in their early twenties, and describes themselves as white, from Austria, and from a working-class family. They have been involved in climate justice activism for 4 years.

Beppo (he/him) is in his early thirties and grew up in a relatively poor family in rural Austria. He receives unemployment benefits from the state. He has been involved in activism for 5 years and climate justice activism for one year.

Rose (she/her) is in her mid-twenties and describes herself as a white Austrian. She is a student, and comes from a middle class, somewhat wealthy family. She has been involved in environmental and climate justice activism for two years.

Sunny (she/her) is in her early twenties. She had a very privileged upbringing in rural Germany. She is currently a student. Her family pays her rent and most of her living expenses. She has been involved in climate justice activism for 4 years.

All participants were extensively engaged in climate justice activism. Many have been arrested repeatedly for their role in direct action, others have worked the equivalent of full-time jobs carrying out unpaid organizational work, while others have lived for extended periods in occupation camps.

## 3. Analysis

Table 1 below provides an overview of the Group Experiential Themes, Experiential Statements, and Example Quotes.

### 3.1. The Earth Is Burning

This Group Experiential Theme contains three Experiential Statements. All relate to the impetus for the respective participants’ engagement in climate justice activism.

#### 3.1.1. Crisis

For all of the participants, there is a deep sense that an immense crisis is taking place. This sense of imminent crisis, and the emotions that it triggers, recurs throughout the participants’ accounts of their activist burnout.

The participants experienced this crisis in different ways and had different names for it. In the below quotes, it is clear how absolutely totalizing and existential the crisis is for Rose.

“*The Earth is burning and everything is so bad*.”—*Rose*

“*The world is going to end and it all sucks. My whole head is just full of crisis. Everything is just one more crisis, everything is wrong. And for me, that… that was just too much.*”—*Rose*

The crisis is so great that it overtakes and eclipses everything else in the world for her, and she cannot think of anything else. It seems that the crisis is something that becomes projected onto everything in her life. She gave an impression of intense emotions like frustration and pain, which was tangible in passages such as these.

The existential nature of the crisis is echoed by other participants. Despite Beppo’s calm manner through the interview, the below quote clearly reflects the urgency of the crisis for him.

“*I see the threat and I also see the pressure to act, which is really becoming more and more urgent*.”—*Beppo*

One has the sense that Beppo is bearing witness to an unfolding catastrophe, which he can see but is not necessarily seen by others. Later in the interview, Beppo goes into more detail about the feelings that the climate catastrophe evokes in him, namely great sadness and grief.

“*It does make me very sad that we are still not able to get a grip on this issue*.”—*Beppo*

“*For me, the climate catastrophe doesn’t create pressure, but rather sadness. Yes, and I have a lot of regret, especially for democracy…*”—*Beppo*

There does appear to be a commonality that to some extent ties these three quotes together: the lack of control that Beppo experiences in relation to the climate crisis.

As the quote below shows, before becoming involved in activism, Hazel tried to suppress their awareness of the really bad aspects of the climate crisis. After becoming involved in activism, the sense of crisis became inescapable.

“*I was actually very optimistic when I joined [social movement]… And then suddenly you find yourself dealing with the climate crisis every day. And somehow, up until then, I just tried a lot to suppress the really bad things so that I wouldn’t have to deal with them too much. And you can’t do that when you’re involved in activism*.”—*Hazel*

The final sentence of this quote shows how, in Hazel’s experience, being burdened by the climate crisis is an inevitable aspect of engaging in climate justice activism.

Several participants used the term “climate anxiety” to refer to this experience and the emotions that it triggers within them. In the below quote, Lilly connects their experience of climate anxiety to the experience of activist burnout.

“*What I also wanted to tell you, also in advance, is that I didn’t just have an activist burnout, but also climate anxiety, and that the two are connected to each other*.”—*Lilly*

This quote occurs very early in the interview; for Lilly this was so clearly relevant, that she brought it up of her own accord. She goes on to describe the feelings that she associates with climate anxiety. Namely, powerlessness.

“*For me, it’s just that I feel a certain powerlessness in everything that happens out there… We actually know how it should be done differently, but then on the other hand this powerlessness. Nobody listens to you, people make fun of you… So, there are people who drive blindly towards the abyss because they simply don’t care. And then there are people who see where we’re going and they also say, “Hey, stop, stop!” And nothing happens. So this feeling of being at the mercy of others and powerlessness*.”—*Lilly*

Again, not only is the existential nature of the climate crisis clear—like driving blindly over the edge of a cliff—but also the totalizing nature of the feeling of powerlessness that it evokes. Lilly feels that she herself is at the mercy of others, placing her into a highly stressful fight for survival. While contemporary system-challenging social movements largely view the climate crisis as a systemic issue that requires systemic solutions, this quote shows how the feeling of powerlessness is experienced at the individual level.

Lastly, Hazel considers whether this sense of urgency is unique to climate activism.

“*I think it’s also just a particular aspect of climate activism that the pressure is much higher. So if you’re doing any other kind of activism, the pressure is probably there too, but it… it doesn’t really… you don’t really have a limit by which you have to have achieved what you want to achieve. And for us it’s kind of like this: what we’re fighting for today should have happened 30 years ago, and that builds up a lot of pressure*.”—*Hazel*

For Hazel, it seems that this absolute urgency is experienced as an intrinsic, inescapable aspect of climate justice activism.

#### 3.1.2. No Alternative to Activism

In their own way, all of the participants view activism as an imperative. The degree to which an individual feels that they have no alternative options open to them is of pressing relevance to the experience of activist burnout.

For Sunny, taking action is tied with avoiding guilt. To engage in activism is to do the right thing, even if she is unable to stop the climate crisis:

“*And I also want to be able to say later on: ‘I did everything I could to make sure it wasn’t as bad as predicted’*.”—*Sunny*

The above quote from Sunny shows how the climate crisis and the need to take action are individualized. In Sunny’s experience, this is a moral imperative that falls upon her as an individual, rather than upon humanity as a whole.

In contrast, Beppo views the responsibility to act as a collective one:

“*We elected these people to get this done for us and represent us in parliament. But now we realize that they are overwhelmed, they can’t do it, it doesn’t work. That’s why this responsibility falls back on us… And now we citizens must all work together to find a new way to shape the transformation*.”—*Beppo*

Nevertheless, action is no less of an imperative for Beppo. The responsibility falls upon us all as a civic duty, and now we must act together.

Other participants are matter of fact and absolute in their description of their internal drive to take action:

“*So I couldn’t just forget about it, because this is my life, all of our lives. And I can’t keep acting like I don’t know that*.”—*Lilly*

For Lilly, engaging in activism is a logical and ethical consequence of her knowledge. Her commitment seems unquestionable, as if she cannot imagine any other way forward.

#### 3.1.3. If Not Me, Then Who?

Again and again, the participants lamented at the shortage of people who were willing to take on commitment. They seemed to feel that the burden of the crisis was theirs alone to carry. In this regard, there are striking similarities in the following four quotes from Rose, Lilly, Sunny, and Beppo.

“*Once you think about it, once you start thinking: everything is going wrong and there are so few of us who are changing anything. And that’s the problem. It’s permanent, we are too few people… There should actually be more, we need more people. Where are the people? Why doesn’t anyone care? Why are there so few people who are doing something?*”—*Rose*

For Rose, it is an ever-present problem; there are permanently too few people. The repeated questions at the end of the quote convey desperation and bewilderment at the lack of people who share the workload, as well as urgency.

“*I asked myself, why is it me doing this, yet again? Why isn’t anyone else doing this? Why are there so few of us? Uff… yeah, it wasn’t a positive spiral*.”—*Lilly*

Just as in the quote from Rose, Lilly repeats the question multiple times. Lilly seems to plead for help, as well as express confusion, disbelief, and bewilderment.

For Sunny, it is clear that the workload cannot be scaled back.

“*A very present topic is, of course, how many people are active. And if we only ever stay at 20 people, then the workload will never decrease because there are so few of us*.”—*Sunny*

She presents the issue in a matter-of-fact way, indicating that for her, the logic is clear: the only option to reduce individual workload is to increase the workforce. If not, then she simply needs to work more.

Lastly, when asked what would have helped him during or leading up to his experience of activist burnout, Beppo responded that he wished for more people to take on responsibility.

“*I often wished that other people would take on more of the responsibility. But… it was, it was difficult, like, there were always a lot of people who wanted to support and get involved, but only very few who really wanted to take on responsibility*.”—*Beppo*

It is significant that Beppo expressed this wish, rather than, for example, wishing that he had not taken on so much work. For Beppo, it is clear that workload cannot be scaled to the size of the workforce, and that there are very few who are “really” willing to take on responsibility. This seems to be a basic truth for all participants in their experience of activist burnout.

There is an undertone of isolation in the above quotes. It is as if the participants are simultaneously saying “I have so much work that I need to do” and asking “why is nobody helping me?”.

Although Beppo states that he does not directly feel the pressure and urgency that many others feel, he is nevertheless indirectly affected by it, as this urgency is deeply ingrained in the working culture of the social movement.

“*The problems were often blown up and people reacted to them in panic mode. And my role was always to say, “okay, we can look at this calmly, we’ll find a solution and bring calm. But that always involved a great deal of effort, because people always wanted to find a solution straight away and react immediately… I have the impression—I can’t look inside people—but I have the impression that the climate catastrophe itself is simply creating this pressure for some people. Which I can also well understand*.”—*Beppo*

Despite efforts to calm the urgency and to engage in his work deliberately, he is greatly strained by the urgency of others and panicked working culture. Although his experience here differs in many ways from that of the other participants, we again see how the atmosphere of urgency pervades activist work and is ultimately inescapable for the participants.

### 3.2. Relationships with Oneself, Other Activists, and the Outside World

This Group Experiential Theme relates to the ways in which the participants’ relationships are characterized by their engagement in activism.

#### 3.2.1. Activism Is My Life

This Experiential Statement describes the participants’ relationship with their activism, as it relates to their own self. This relationship can be characterized as all-consuming, almost as if the participants lose themselves within their activism. This type of Experiential Statement was the most common among all of the participants, which seems to indicate its importance.

The following quote from Beppo succinctly summarizes how he experiences this phenomenon:

“*Actually it was like a full-time job… Actually, it wasn’t like a job, instead it filled up my entire life*.”—*Beppo*

It seems that Beppo is talking about something more than the great quantity of time invested. His activist work spills over into his life in a much more personal way.

In the following quotes from Sunny and rose, we see further examples of the deep enmeshment between private life and activism, and difficulty with locating the point of separation.

“*So my free time is my activism. And my activism is my free time. I don’t really have many hobbies, almost none*.”—*Sunny*

“*It’s always so difficult for me—what is it? Is it like work, is it like a hobby, is it like a private life? Somehow it’s a mixture*.”—*Rose*

Tension is evident in the quote from Rose; it is a difficult topic for her—she is not sure of the role that activism plays in her life. Although she does answer her own question, it seems that the answer is not entirely satisfactory, rather, it is tentative.

Repeatedly, participants alluded to being out of touch with their own personal boundaries in the moment, and only retrospectively realizing that they had overstepped their own boundaries:

“*It was precisely because I saw how much there actually is to do and where things need to be done that I got into activist burnout, because I didn’t say no to anything*.”—*Lilly*

In this quote from Lilly, activist burnout is a consequence of never drawing personal boundaries. It seems that, in retrospect, she views this to have been her responsibility on an individual level for not having drawn boundaries.

Similarly, Rose attributes the inability to recognize or draw personal boundaries as the cause for activist burnout:

“*The reason for activist burnout is simple: we give ourselves our tasks. And nobody says ‘that’s enough’, but instead we keep giving ourselves tasks. Unless you have a really supportive circle of friends, of course, but we are our own bosses and there is no maximum capacity or time that you invest. So you actually need boundaries*.”—*Rose*

In order to assert boundaries, one must first know where those boundaries are; one must first be in touch with one’s self and one’s emotions. For Rose, this was not the case:

“*And it was only then that I realized that I had no relationship at all with my own feelings… I reflected on what I had actually done and that I had actually, from one moment to the next, thrown my whole life away*.”—*Rose*

Rose underscores the severity of her alienation from her own feelings; they are only something that she can grasp in retrospect. Coupled with the way in which activism has subsumed her life, the consequences of crossing her own boundaries are immense for Rose. In her understanding, she has thrown away her whole life.

Similarly, Sunny states that she lacks an awareness of her own boundaries and capacities in the moment:

“*So for me, it’s very often that I realize in retrospect that it was too much. It’s not at the moment that I realize it’s too much*.”—*Sunny*

In Hazel’s experience, excessive workloads within the social movement are part and parcel of the work culture:

“*I think it probably would have helped if the overworking had been less frequent in my circle of friends and in [the social movement]. It was just a very everyday thing, also that you were partly bragging about how much you worked. It was very normalized*.”—*Hazel*

Hazel apparently felt some competitiveness in the amount of work that they took on. In their experience, overworking is something to feel good about. When we couple this statement with a later quote from Hazel, it seems that they derived a significant degree of self-worth from their activist engagement:

*Hazel:* “*and it was like, it was really obvious to other people that I was completely overworked, but not yet to me, that people often said, ‘hey, don’t do so much, I can take things off you’ or something, but I didn’t want to hear that at all. So I don’t know if it would have helped if that had happened more often*.”

*GL:* “*Why didn’t you want to hear that?*”

*Hazel:* “*Because I think I somehow defined myself 100 percent by what I do*.”

For Hazel, their identification with activist work essentially made it impossible for them to accept the concern voiced by their activist colleagues. Likewise, if self-worth is connected to one’s activist work, then failure can be devastating:

“*And then I really know: I have to stop because it only brings negative feelings. I think I’m bad. I think everything is bad*.”—*Rose*

In that moment, Rose does not view herself as a bad activist, or having failed at a task, but rather as intrinsically bad, as she seems to closely identify with her activist work. Boundaries are blurred and being a bad activist means being a bad person.

We will end this section with a retrospective quote from Lilly, who compares her earlier personal sacrifices to her present efforts at drawing boundaries:

“*I have sacrificed myself. Yes, I’m actually not doing that at the moment. At the moment I’m very much about ‘fuck it’ and I’m doing what I enjoy, because I can’t do [activist] organizing unless I enjoy it*.”—*Lilly*

Note that Lilly does not say that she sacrificed her time, her friendships, or her money. Rather, she seems to have confused her activist work with her very self.

#### 3.2.2. My Relationships with Other Activists Are Complicated

The relationship with other activists is one of the most complex and varied aspects of the participants’ experiences. At times, relationships with other activists can be a support; at other times, they are a burden. The bond between activists can seem robust and stable, and at other times fragile. In all cases, the participants seemed to assign significance to these relationships in their experience of activist burnout.

This apparent ambivalence is evident in the following quote from Rose:

*Rose:* “*I want to go to the plenum every week so that I’m part of the group. And if I haven’t been to a meeting since July, like it is now, I haven’t seen people for weeks. I’ve lost the relationship. So I have no more friendship, no more connection. And it’s so hard to stay in touch with people*.”

*GL:* “*When you’re not there?*”

*Rose:* “*Yes, because even in activism there is so little time for private life. There is no time for private life. I talk to people, I work with people, but I don’t know what they do in their lives. I don’t know what their hobbies are, I don’t know what their worries are, what their dreams are. I don’t know what their private life is*.”

Rose wants to be part of the group, wants to be connected to the other activists, but the only way that she can do that is through engaging in activism, because it is the only thing that they have in common. Rose goes on to describe how the desire for closeness with other activists directly and negatively impacts her recovery from activist burnout:

“*I feel like I just want to go there now so that I can be part of the group, because I miss them, because I want to be part of the group again and I want to have those relationships again. But that’s stupid [laughs] because it stresses me out at the same time*.”—*Rose*

Rose finds herself in a dilemma here; she longs for closeness with the other activists, but it seems that she knows this is likely not possible. Stopping involvement in order to look after her health, means facing further isolation.

In the following quote from Hazel, there is a very similar tension between a desire for closeness with other activists and withdrawing from activism in order to recover:

“*I didn’t do anything for two months and then slowly tried to get back into it, because at the time activism was the main reason why I came out of my burnout in the first place. Not very healthy [laughs]… and also the people, because I really liked them and wanted to see them again*.”—*Hazel*

Notice that for Hazel, maintaining relationships with the other activists was a major part of the reason to return to activism. As is the case with Rose, Hazel faces a dilemma: they want to quickly return to activism out of a desire for closeness with the other activists, but this is in conflict with their health.

We have seen how some of the participants experience friendship in the context of activism. Let us now look at the issue of working together through two quotes from Sunny and Beppo:

“*And a friend of mine, for example, has sort of—I found it quite interesting—expressed the opinion that social movements work a bit like a political party, that you need to convince other people to work with you on a project*.”—*Sunny*

This quote displays another side to the relationship with other activists. Sunny’s metaphor gives the impression of strategically engaging in interpersonal relationships. Being a successful activist requires engaging in this game of internal politics. It seems that, for Sunny, the relationship with other activists is at least partially characterized by competition and unspoken power dynamics.

Beppo also feels the need to strategically engage with other activists:

“*And then, let’s say, you always went into the meeting already knowing that ‘okay, she’s sure to have this position again, and he’s sure to have that position again. There’s bound to be a conflict’ and then it was often a tactic at play to try to… convince individual people of your arguments in advance. And there was simply too much pressure involved*.”—*Beppo*

Note Beppo’s word choice: “position”, “tactic”, and “arguments”. To some degree, it seems that activist work requires instrumentalizing his relationships with other activists. However, the need to strategically enter into social relationships is in tension with the idea of friendship-like bonds between activists, which seems to create considerable tension for Beppo.

Lastly, the relationship with other activists can also be a source of support. When Beppo had to leave his role in the social movement at short notice (due to activist burnout), he received understanding and appreciation:

“*Everyone was very understanding and many people expressed their appreciation and gratitude. That was very nice*.”—*Beppo*

For Beppo, it seems that receiving this support from the others was very important. His use of superlatives gives the impression that he experienced an outpouring of support, which made it much easier for him to leave his role within the social movement with a good conscience.

Although support from other activists can be meaningful, it can also be fragile:

“*So I was, I felt like, I was alone and I realized: I invested so much and I got nothing. So I thought to myself, I was with so many people every day. And now I’m alone. Where are the people? Where is the network? Where is this community that helps me when I’m not doing well?*”—*Rose*

After the occupation was evicted, Rose was cut off from the community of other activists. In this case, the community was broken up through state repression. After investing so much in these relationships, they seem to evaporate instantly, and Rose seems to feel isolated and bewildered.

Sometimes, the need to support others in the group can itself be a burden:

“*I’m always afraid that if people know, they’ll feel like they can’t give me any more to-dos. And then I’m afraid that they’ll do too much because they know that I’m doing badly. But still, it’s important to me that other people know they can still rely on me, even if it’s too much for me at the moment*.”—*Sunny*

In this quote there is a tension between Sunny meeting her own mental health needs and continuing to carry her own portion of the workload to ensure that others do not become overburdened. Here, being reliable for others takes precedence over her own needs.

As we have seen in this section, relationships with other activists are heavily characterized by the activist work itself, are anything but one-dimensional, and can simultaneously be both a resource and a stressor within the experience of activist burnout.

#### 3.2.3. Activism Characterizes My Relationships to the Outside World

In general, the participants’ relationships with the world (and people) outside activism are characterized to a large degree by isolation or distance, which can extend to anger, frustration, or an “us-against-the-world” mentality.

“*I felt a bit like I and the movement were the only ones who realized that the world was coming to an end. Nobody listens to me and I have to kind of scream and scream and work and work and work to get people to realize what is going on*.”—*Hazel*

Here Hazel draws a stark contrast between those who understand the gravity of the climate crisis and those who do not. On the one hand, Hazel identifies themselves strongly with the social movement, while everyone else is a source of frustration and isolation for Hazel. It seems that Hazel feels forced into a passive, victim role by people who do not understand that the world is ending.

The following quote from Sunny shows how tension is possible even when encountering people who may be sympathetic to the social movement.

*Sunny:* “*They don’t even know what they can do and then say—I hear this very often—this ‘oh, it’s very important that you are so involved, thank you’, but it’s not an ‘oh, how can I also get active’, so I think that’s an external factor that always plays a bit of a role and that also comes back to this inclusivity, effectiveness*.”

*GL:* “*What is it like for you when you have these encounters? What does it do to you?*”

*Sunny:* “*A sort of anger, because it’s very convenient to pass the work on to others*.”

It seems that Sunny also feels forced into a passive, victim role. In this passage, it is as though activist work is being forced on to her by people outside the movement who are not willing to help. This evokes anger and resentment in Sunny.

Some participants describe how this tension affects close interpersonal relationships. In this passage, Lilly describes how her activism has alienated her from some of her closest friends:

“*…for me, it went so far that I even made my closest friends feel guilty because they were driving, or because they were transporting things by car, or just… for me they were always doing much too little*.”—*Lilly*

It seems that Lilly wants to emphasize to the researcher the extreme degree to which her personal relationships were affected. She does not describe herself as the victim of others’ actions. Instead, she is the one who acted aggressively. One wonders if, in reality, her friends would ever have been able to live up to the standard that Lilly expected of them. For her, their actions were always far too little. Later in the interview, Lilly speaks of the guilt that she used to feel in connection with her own actions (for example, when she bought a plastic bottle of water). In this context, the guilt that she evoked in her closest friends likely had much to do with her own feelings of guilt, as well as the unreachable moral standard that she held herself to.

Some of the participants described how their relationship with those outside the movement impacts their experience of seeking mental health support. Below are two passages from Rose, one describing her wish for a self-help group for activists and the other describing her experience in psychotherapy.

“*I have this little dream of a support group for activists. Because it’s just not understood… I just don’t feel understood by people who aren’t going through it themselves. I have a problem comparing it to a teacher’s burnout or something. It’s just a special situation, I think, that you can’t easily compare with other things*.”—*Rose*

“*If I then tell a therapist who says, maybe you just have to… no, don’t drop out of university, think about what you want to do in your future. And I’m like “what work, what work?” I work, I don’t get paid for it yet. I don’t need it. So which world do I want to work for? What world do I want to make money in when the world ends? So I don’t need work. So it’s this radicalism, this understanding of the world, that many people don’t understand. You have to have the understanding and the view of how the world works. Or when I talk to my colleagues now, they don’t understand. For me, things are normal that are simply not on the radar for other people, they don’t think about it and I just don’t feel understood*.”—*Rose*

Both passages show the ways in which Rose feels misunderstood by those who do not share common ground with her, either in terms of activist burnout, or in terms of a radical world view. A shared reality is a must for Rose. She seems to be saying that the solution to her activist burnout must come from within the world of activism. As we saw in the previous section, a desire for closeness with other activists—her little dream of a self-help group for activists—is the counterpart to her perceived isolation from the world outside of activism. For Rose, her problems are so interlinked with the climate crisis that it is not possible to understand one without the other.

### 3.3. Burning out

While the previous sections describe the participants’ experiences of burnout in a broader sense, this section looks more closely at the experience of burnout when it felt the most acute for the participants.

#### 3.3.1. Cynicism, Stress, and Failure

The following passages provide a closer account of the emotional “symptoms” that the participants experienced, which were primarily exhaustion, stress, cynicism, and a loss of motivation.

In the following quote, Lilly describes how activist work became a joyless endeavor for her:

“*I think I always had a lot of fun organizing and mobilizing and all that. But because I did it too much, I lost the fun of it, you know? And then it just wasn’t fun anymore. It was all just a pain in the ass. It was so exhausting. It was just nerve-wracking*.”—*Lilly*

The joylessness became absolute for Lilly; a central part of her identity, from which she took great pleasure, becomes nothing more than nerve-wracking, strenuous, and joyless. Moreover, there is a sense that this cynicism spilled over into the rest of her life; at the end of the quote, it is not clear whether her feeling of cynicism and joylessness is experienced only in relation to activist work.

In the following quote from Sunny, the phrasing evokes a feeling of spiraling anxiety.

“*In my head there’s a lot going on and all at the same time. So I also notice that when I try to take a break and really, I don’t know, lie on the couch and do less and let my brain calm down a bit, it’s still… the brain doesn’t calm down because it’s constantly like ‘okay, I still have to write this to-do down so that I can do it the next day or I still have to do this to-do today or I still have to tell that person this’*.”—*Sunny*

Whenever Sunny tries to take a break and close her eyes, thoughts about activist work swirl around in her head. It is as if Sunny has the feeling that if she can just check off the tasks that she has to do, then she will be able to relax. At the same time, it seems that no amount of work will satisfy her. There is no ‘end’ in sight. For Sunny, it seems that activist work is like a loose tooth that one cannot stop probing, or an itch that one cannot seem to scratch.

Lastly, we look at two accounts from Beppo and Rose that more closely describe a feeling of cynicism. In the following two passages, Beppo describes how he became cynical about the tactics of the social movement. As a result, he was no longer able to balance out the stress and pressure connected with his activist work.

“*I doubted more and more whether this confrontational form of protest was really the right thing for me… That was actually my last appearance, at a protest… And then the motivation was completely gone for me… my motivation to continue was gone and that’s why I left*.”—*Beppo*

“*I was no longer able to deal with the stress and pressure because I no longer really believed in it. How can you put it? It’s… like a scale. If you have a lot of conviction and a lot of motivation, then you have no problem at all with stress and pressure. Yes, of course you can’t do it for a very long time, but you find a way. But if your motivation decreases, your conviction decreases, and your doubt increases, then you can no longer cope with the pressure and stress. Then your energy will go down and down*.”—*Beppo*

In Beppo’s experience, motivation and conviction are the counterweight to stress and pressure, at least temporarily. Interestingly, he draws a one-way relationship between a loss of motivation, and subsequently succumbing to stress, pressure, and exhaustion. If he had only believed in the tactics, it seems, then he would have been able to hold out against the pressure, at least for a while longer. Nevertheless, it seems that for him there was always a degree of inevitability in terms of his giving in to stress and pressure.

In another passage, Rose expresses cynicism looking back on her activist engagement:

“*I gave up everything just for this. And now it’s over. So I’ve actually given up my whole life for nothing, because it’s over. It achieved nothing*.”—*Rose*

Rose’s cynical outlook, as well as her feeling of absolute loss, is tangible. She gave up everything—her entire life—for nothing. The degree to which she identifies with the social movement is expressed as absolute. If the social movement collapses, then the time and energy that she has invested into the social movement are futile. It seems that for her, failure and success are binary. Furthermore, she seems to identify strongly with the social movement—the movement has achieved nothing, and by extension, she has achieved nothing. At least in this passage, it seems as though Rose does not have perspective regarding the future, evoking a feeling of loss and hopelessness.

#### 3.3.2. The Last Straw

Activist burnout seems typically to be a longer process, like water that heats up before coming to a boil. Nevertheless, for almost all of the participants, there was a concrete “tipping point”.

For some this was coupled with a somatic condition. In the following passage, Sunny describes how her body gave out as a response to the stress and pressures of her activist work:

“*I woke up at night and went to the bathroom and collapsed on the toilet. I fell against the wall and busted open my lip. And then I actually thought, ‘okay, now my body is telling me very clearly to stop’… And then we actually went to the hospital… And they didn’t find anything. Everything was okay. It was just psychosomatic, the stress that was so bad for my stomach. So it was these physical symptoms of ‘too much’, so to speak…*”—*Sunny*

We see how sudden and acute this moment was for Sunny, a moment of literal collapse into unconsciousness, which comes on unexpectedly during a vulnerable moment—at night while alone in the bathroom.

In the account of activist burnout from Beppo, a somatic condition contributed to his lack of energy and motivation, leading up to the “tipping point”:

“*But I stepped away more and more and did less because I didn’t have any energy. I was just… I just didn’t have the motivation… And then I also had to take antibiotics, but not for anything serious, but then my digestion was broken. The flora was destroyed and it took weeks to recover… On top of that, I had very little energy left. But I still got quite a lot done… And then I went on vacation… I was able to relax very well and take my mind off things. I came back with more energy. Then I worked full-time again for just one day, with meetings and answering e-mails and questions and so on. And then in the evening I was totally knocked out again and had no energy at all and then the next day I didn’t feel like continuing at all. And then I made the decision and said I’d stop now. That’s what happened. A real lack of motivation, a crisis of motivation*.”—*Beppo*

In this passage, Beppo does not seem to attribute the somatic condition to the stress associated with his activist work. Nevertheless, the condition seems to play a pivotal role in his burnout. Despite his vulnerable condition, the actual “tipping point” comes some time later, upon returning to his activist work after a holiday. At first glance this appears paradoxical. He is able to relax during his holiday, and returns to activism with more energy, but one full day of work leaves him once again totally knocked out.

To obtain a more complete picture, we return to an earlier statement from Beppo, in which he describes the period leading up to the holiday he describes above:

“*Then I just carried on, even though I doubted our work, because I knew that it was necessary that… there were no people who could take on this task. And then there were several people in my [team] who did it very well and I had the feeling that I could now leave without causing a big problem*.”—*Beppo*

It seems that for Beppo, the holiday was confirmation that the social movement can continue without him. Until that point, he had been able to sustain his work through a feeling of responsibility and necessity, but once this fell away, his body and psyche were able to end the stress cycle and resisted returning to work. It was no longer necessary for Beppo to push himself to the limit.

Hazel also experienced a tipping point that coincided with a holiday:

“*I slowly realized that it was getting too much for me and that I needed to withdraw. And then I almost went through with it. And then came two very emotional… things. One was that the [city] threatened to sue some of us and me for millions because of our activism. And that was very emotionally stressful… Then it was the Christmas vacation and I realized that I couldn’t recover. I didn’t do anything for a week and then I wanted to study for exams again. I realized I just couldn’t do it. And then at the occupation, an arson attack happened. So probably Nazis set fire to a wooden hut while people I knew were inside. And nothing happened to anyone, but it was just very emotionally stressful. And I think that somehow felt like the ‘last straw’*.”—*Hazel*

Here, we see how Hazel realized that they were near the point of burnout but felt that they were unable to withdraw their involvement due to the consequences of activism. The existential nature of these consequences meant that taking a break was difficult or impossible. After this ‘last straw’, Hazel experienced physical exhaustion over a period of several months.

In her account, Rose identifies the point that pushed her over the tipping point: the eviction of the occupation that she was involved in.

“*The thing that, ah, got me down was really this ‘it’s over’ situation. So for a year, every week plans, every day camp, all the same people, actions, always this one theme, then from one day to the next it’s over. The place gone, the people gone, the idea gone, the plans gone, no strategy, nothing, everything gone. That was so, so bad for me…*”—*Rose*

From this passage, it is clear what a sudden blow the eviction was for Rose. As we have seen in other passages, Rose experienced the eviction as a loss that was absolute—everything was gone, and the project or movement was over. After all the work that she put in, she was left isolated, with nothing to show for it. At the end of the passage, she switches from past to present tense, which seems to indicate how immediate the topic remains for her.

In a later passage, Rose is speaking about a lack of acknowledgement for the gravity of consequences in activist culture. Near the end of the passage, she refers again to the eviction:

“*And that’s… this acknowledgement that these are difficult situations that burden us. I don’t think that happens enough. I think there’s a lot of ‘yes, it doesn’t matter and we’re all used to it and yes, it’s normal’. It’s not normal. Normal people are not arrested. Normal people don’t live in occupations and then everything is destroyed. So that’s not normal for me. It’s an extraordinary situation that is extremely stressful for the psyche*.”—*Rose*

Again, we see how absolute this loss was for her: “and then everything is destroyed”. Rose draws a contrast here. Although defeat is a “normal” aspect of activism, the gravity of the consequences of activism are not so easily integrated into her psyche. Rather, these experiences are extremely stressful. In both of the above passages, there is an undertone of isolation, whether in terms of the support structures and relationships that fall away with the eviction, or a lack of understanding within the social movement for the difficulties she is facing.

## 4. Discussion and Implications

### 4.1. Summary of Results

The results provide a largely coherent picture of activist burnout among the participants, which is characterized by a sense of imminent and inescapable existential crisis. This is connected to feelings such as powerlessness, desperation, sadness, frustration, anger, or guilt, and lends a pressing sense of urgency to the individual’s activist work. The individual sees activism as a logical consequence, something that simply must be carried out and for which there is no alternative. The sense of urgency and inevitability translates to a relentless work ethic, in which there is always a shortage of others willing to take responsibility.

Either gradually or abruptly, the individual leaves behind their ‘private’ life. Friends, family, hobbies are neglected. Activism may become a major part of their identity, consume their thoughts, become connected with their self-worth, and give them a sense of purpose.

The relationship with other activists is complex. They form the individual’s primary social circle. The individual may face a dilemma: if they step away from activism, they may lose these relationships. Other activists may not function as an effective support network. The individual is prone to frustration with their activist colleagues, or to feelings of competition and insecurity. Meanwhile, the individual experiences a degree of alienation from the outside world. They may feel that others do not understand their world view and life choices or feel resentful towards non-activists.

The individual may have a degree of awareness that they are burning out. They have likely been cautioned by concerned friends or other activists but have not been able to heed the warning signs. Indeed, they may have viewed burnout as inevitable, as if they knew it was “part of the game” all along.

Activist work, once a source of meaning and identity, becomes joyless and anxiety-inducing. The individual is trapped between the need to maintain control over their activist work, and the need to take care of themselves. They may experience a spiral of anxiety avoidance in relation to their activist work. The individual may become bitter, pessimistic, and inflexible in regard to their activist work.

As the activist burnout develops, the individual may experience a moment of clarity, in which they are confronted with the reality of their situation. If not, they may reach an acute “tipping point”, where the scales shift and it is no longer possible to continue. For some, this moment is accompanied by a somatic or psycho-somatic condition or experienced as complete exhaustion.

### 4.2. Relation to the Literature

The way in which activist burnout was experienced among the participants appears to have much in common with descriptions of burnout among the helping professions. In this literature, emphasis is placed upon the motivation that drives the individual to engage in their work. Within the helping professions, this is often a desire to ‘help’, which may be idealized as selfless or altruistic dedication [25] (p. 69). Freudenberger [4] originally described the most burnout prone individuals as those who are dedicated and committed, who feel pressure to help from within. This description would seem to fit many of the participants within the present research.

The dynamic of engaging in ‘altruistic’ work in a quest to satisfy personal needs, and subsequently overburdening oneself, has been described by Maslach [25], Freudenberger [4], and Schmidbauer [6]. Interpreting the results in this regard requires some caution, but we have seen that for some participants, engaging in activist work was a way to deal with their own guilt or anxiety connected to the climate crisis, while others derived identity and self-worth through their activist work.

Maslach [25] notes how, among many people who burn out in the helping professions, “helping people is not viewed as just a job but as an expression of one’s personal identity” [25] (p. 70). In relation to activist burnout, Gorski [15] found that participants tended to view their activism as a “calling”. When the role of rescuer or helper becomes entwined with one’s personal identity, it becomes very difficult to maintain adequate distance or reduce workload. In some ways, the participants in the present research take upon the ultimate rescuer role: saving the world, democracy, and the global south. It certainly seems that many participants view activism as a “calling”, a predestined path that one is unable to steer away from. In the context of climate justice activism, it seems that the activist’s identification with their activism and their sense of purpose has a complex interaction with activist burnout.

In their research on burnout in social justice and human rights activists, Chen and Gorski [3] described “deep sensitivities to injustice” as one of several specific causes of activist burnout. The present research suggests that the phenomenon of activists being overwhelmed due to “sensitivity” to injustices may be better described as an issue with maintaining boundaries and distance. “Sensitivity” is not necessarily the problem. In fact, Maslach [25] (p. 14) describes emotional detachment, i.e., depersonalization, as a symptom of burnout. What is necessary is the ability to exercise awareness, regulate intense emotions, and to adequately distance oneself. According to Sendera and Sendera [26], the ability to distance oneself from one’s work is the primary factor in burnout, and describes why some do burnout while others do not, even within the same work environment.

Freudenberger’s [4] account of burnout arising when the individual is pressured by their boss to give even more—as well as Sendera and Sendera’s [26] description of burnout in the context of structural/hierarchical power dynamics—gives rise to the question of whether there is a parallel amongst the participants in the present research. After all, the participants in the present study did not refer to being negatively affected by coercion and power dynamics within their social movements. On the one hand, activist work within teams poses challenges, even when hierarchies are relatively flat. Previous studies on activist persistence and activist burnout have found that “in-movement” causes of burnout (e.g., in-movement conflict or cultures of martyrdom) are common [13], and the results of the present study seem to reflect this.

The results of the present research show that the relationship with other activists is complex in the context of activist burnout. Previous research shows that interpersonal relationships within social movements can be a supportive factor that counterbalances the factors leading to activist burnout [2]. However, the results of the present research show that these relationships can also be a considerable source of stress, for example, when they take on a competitive dimension, when conflicting viewpoints arise, or when they fail to provide deeper closeness and emotional support. Additionally, activist structures can be fragile and transient; activists come and go, and social movements have a lifecycle [27].

Broadly speaking, the symptoms of activist burnout documented within the present research mirror, but also significantly expand upon, descriptions of activist burnout within the literature. Previous research noted a general deterioration in psychological well-being, including depression, stress and panic attacks [3], feelings of isolation and hopelessness [15], and compassion fatigue [12], as well as (psycho-)somatic conditions such as exhaustion, migraines, sleep disorders, and cancer. In the context of burnout among the helping professions, Maslach [28] described three dimensions of burnout symptoms: exhaustion, cynicism (or depersonalization), and inefficacy (or reduced personal accomplishment).

The present research lends a more complex, rich account of these symptoms among climate justice activists. For example, many of the participants described cynicism towards their activist work—their activist work became joyless, or they lost conviction in the tactics or goals of the movement. In some cases, this cynicism extended to interpersonal relationships both within and outside of the social movement. Many participants reported feelings of low self-worth and of self-doubt, which seemed to be connected with their identification with activism and feelings of inefficacy. The anxiety-avoidance and procrastination spiral described by several participants has been noted in the literature on burnout [26]. Amongst the participants, the lack of distance from activist work is perhaps most clearly visible in the phenomenon of not being able to “turn off” the stream of thoughts connected to activist work.

The results show that activist burnout cannot simply be reduced to an individual self-care issue. This is corroborated by the academic literature on activist burnout and burnout within the helping professions. Gorski [29] found that mindfulness practice had positive mental health effects for activists but called upon leaders in organizations and movements to effect cultural change in order to mitigate activist burnout. Vaccaro and Mena [12] found that activist knowledge of self-care practices was not sufficient to prevent negative mental health outcomes. Conner et al. found that self-care is particularly challenging for activists who tend to be “motivated from a place of selflessness” [2] (p. 12).

### 4.3. Strengths and Limitations

Due to its exploratory and idiographic nature, using a relatively small sample size, the present research cannot make claims about activist burnout at the population level. It is not intended to test a hypothesis or theory. Instead, the present research is a starting point for theory formation regarding the experience of activist burnout, specifically among climate justice activists.

Furthermore, although there is a degree of diversity amongst the participants in terms of age, socio-economic background, and gender identity, there is a lack of ethnic or racial diversity. The researchers suspect that this mirrors the demographics of the climate justice movement in the global north and central Europe in particular.

According to Smith [21], for IPA studies with a sample size of five, each Experiential Statement should be supported by extracts from at least three participants. Within the present research, each Experiential Statement is supported with extracts from four participants, with the exception of “Activism is my life” (Section 3.2.1), which is supported with extracts from all five participants, and “No alternative to activism” (Section 3.1.3), which is supported with extracts from three participants.

In regard to demographics, the main researcher/interviewer can be described as US-American WASP (white, Anglo-Saxon, protestant, male). Although effort was taken to reflect upon bias, the researcher’s standpoint undoubtedly affects the research data collection and analysis. One potential way to limit this effect would have been to use a team of researchers to collect the interview data; however, this was not possible at the scale of this research. The collection of data by one researcher does have its own benefits—having engaged face-to-face with the participants, the researcher has a more detailed and nuanced impression of their accounts.

### 4.4. Implications for Activists

The activist should carefully and regularly consider the reasons that they are engaged in activist work, and how activism interacts with their identity. Identifying too rigidly with an activist role can make it difficult to heed personal boundaries. Activist work cannot satisfy all emotional needs. It cannot replace self-care, hobbies, or relationships, and it cannot fill emptiness. Activist work is work, and work needs to be kept within balance. Likewise, relationships within social movements can be meaningful, but they can also be transient and fragile.

If one feels a need to ‘martyr’ oneself, carefully consider the reasons why.

The activists should consider how they frame success. Is it possible to be successful without effecting radical change, or is there no such thing as ‘enough’?

Lastly, self-knowledge is necessary for avoiding burnout and engaging in activist work in a healthy and sustainable way.

### 4.5. Implications for Organizers and Practitioners

Burnout is a multidimensional process, meaning that organizations share in the responsibility to prevent burnout. As described above, activist burnout among climate justice activists appears to have much in common with burnout within the helping professions, where supervision and other processes for reflection at the individual and team level are seen as a necessary part of the job. Leaders and organizers within social movements, as well as practitioners within the helping professions, have a duty to mitigate and treat negative health outcomes within this underserved demographic. This could take place through cultural change and processes for reflection within social movements, as well as through targeted support for activists, e.g., through supervision or therapeutic workshops.

### 4.6. Implications for Researchers

There is a paucity of research evaluating the effectiveness of burnout interventions in general [7], and the field of activist burnout is no different. One potential opportunity is to action research and case studies on the topic of supervision and mental health support within social movements. It should also be taken into account to potentially screen participants at baseline for potential character traits, strength, weaknesses, or other personality characteristics, which should be performed in a participative manner [24].

## 5. Conclusions

This exploratory, qualitative study examined how climate justice activists make sense of their activist burnout. Semi-structured interviews were conducted with five climate justice activists (*n* = 5) who are active in Austria. The interview data were analyzed using Interpretative Phenomenological Analysis.

The participants’ experiences of activist burnout can be characterized by a persistent sense of crisis and pressure, a relentless work ethic, blurred boundaries in regard to work, complex and sometimes ambivalent relationships with other activists, and a degree of alienation from non-activists. When their burnout was most acute, they experienced cynicism, stress, and exhaustion, as well as an abrupt “tipping point”.

Parallels were drawn with descriptions of burnout within the helping professions, particularly in regard to identification with work and the pursuit of work to satisfy personal needs. Activists are recommended to examine the reasons why they engage in activism. Organizers are reminded that the solutions to activist burnout cannot be individualized. Researchers are asked to investigate the efficacy of interventions for activist burnout.

## Figures and Tables

**Table 1:** Overview of Group Experiential Themes and Experiential Statements.

Group Experiential Themes	Experiential Statements	Example Quotes
(Section 3.1) The earth is burning	(Section 3.1.1) Crisis	“*The Earth is burning and everything is so bad*.”*—Rose*
	(Section 3.1.2) No alternative to activism	“*I couldn’t just forget about it, because this is my life, all of our lives. And I can’t keep acting like I don’t know that*.”*—Lilly*
	(Section 3.1.3) If not me, then who?	“*Where are the people? Why doesn’t anyone care? Why are there so few people who are doing something*?”—*Rose*
(Section 3.2) Relationships with oneself, activists, and the outside world	(Section 3.2.1) Activism is my life	“*Actually it was like a full-time job… Actually, it wasn’t like a job, instead it filled up my entire life*.”*—Beppo*
	(Section 3.2.2) My relationships with other activists are complicated	“*It’s important to me that other people know they can still rely on me, even if it’s too much for me at the moment*.”*—Sunny*
	(Section 3.2.3) Activism characterizes my relationship to the outside world	“*I felt a bit like I and the movement were the only ones who realized that the world was coming to an end*.”*—Hazel*
(Section 3.3) Burning out	(Section 3.3.1) Cynicism, stress, and failure	“*I was no longer able to deal with the stress and pressure because I no longer really believed in it*.”*—Beppo*
	(Section 3.3.2) The last straw	“*And then I actually thought, ‘okay, now my body is telling me very clearly to stop’*.”*—Sunny*

## Data Availability

The data presented in this study are available on request from the corresponding author.

## Abbreviations

The following abbreviations are used in this manuscript:

IPA	Interpretative Phenomenological Analysis

## Appendix A

	**Interview Guide**
1	To the extent that you are comfortable with, please provide the following demographic and background information:
	1. Ethnic or racial identity
	2. Socio-economic Status
	3. Gender identity
	4. Age
	5. Years of experience in activism
	6. Types of activism that you are involved in
2	How did you come to be involved in activism?
3	What are the different aspects of activist work?
4	Tell me about the aspects of activist work, which you find the most difficult.
5	Tell me about a time when you were particularly under pressure due to your engagement in activism.
6	Tell me about a time when you had to withdraw from activism.
7	Can you describe what the period of activist burnout was like for you?
8	How has it gone for you since then?
9	What helped you?
10	How would you have wished for things to go differently?
11	Is there anything else that you would like to share with me?
